# Cost-effectiveness of a new strategy to identify uncomplicated gallstone disease patients that will benefit from a cholecystectomy

**DOI:** 10.1007/s00464-016-5256-4

**Published:** 2016-09-21

**Authors:** Mark P. Lamberts, Cihan Özdemir, Joost P. H. Drenth, Cornelis J. H. M. van Laarhoven, Gert P. Westert, Wietske Kievit

**Affiliations:** 1 Department of Gastroenterology and Hepatology, Radboud University Medical Centre, PO Box 9101, 6500 HB Nijmegen, Nijmegen, The Netherlands; 2 Department of Surgery, Radboud University Medical Centre, Nijmegen, The Netherlands; 3 Scientific Institute for Quality of Healthcare (IQ Healthcare), Radboud University Medical Centre, Nijmegen, The Netherlands; 4 Department for Health Evidence, Radboud University Medical Centre, Nijmegen, The Netherlands

**Keywords:** Cholecystectomy, Watchful waiting, Gallstone, Cost-effectiveness, ICER

## Abstract

**Background:**

The aim of this study was to determine the cost-effectiveness of a new strategy for the preoperative detection of patients that will likely benefit from a cholecystectomy, using simple criteria that can be applied by surgeons. Criteria for a cholecystectomy indication are: (1) having episodic pain; (2) onset of pain 1 year or less before the outpatient clinic visit.

**Methods:**

The cost-effectiveness of the new strategy was evaluated against current practice using a decision analytic model. The incremental cost-effectiveness of applying criteria for a cholecystectomy for a patient with abdominal pain and gallstones was compared to applying no criteria. The incremental cost-effectiveness ratio (ICER) was expressed as extra costs to be invested to gain one more patient with absence of pain. Scenarios were analyzed to assess the influence of applying different criteria.

**Results:**

The new strategy of applying one out of two criteria resulted in a 4 % higher mean proportion of patients with absence of pain compared to current practice with similar costs. The 95 % upper limit of the ICER was €4114 ($4633) per extra patient with relief of upper abdominal pain. Application of two out of two criteria resulted in a 3 % lower mean proportion of patients with absence of pain with lower costs.

**Conclusion:**

The new strategy of using one out of two strict selection criteria may be an effective but also a cost-effective method to reduce the proportion of patients with pain after cholecystectomy.

Gallstones constitute a significant health problem in developed societies, affecting 5–22 % of the adult population, but only an estimated 13–22 % of gallstone carriers will eventually become symptomatic [1, 2]. The diagnosis of uncomplicated symptomatic gallstone disease is based on the Rome III criteria consisting of a steady abdominal pain, usually located in epigastrium and/or right upper quadrant lasting 30 min or longer in the presence of radiologically detected gallstones [3, 4]. However, the sensitivity of these criteria is limited and 40 % of the patients with symptomatic gallstones report far less specific abdominal pain symptoms [5, 6].

A cholecystectomy is the therapy of first choice for patients diagnosed with uncomplicated symptomatic cholecystolithiasis [7]. There are no international guidelines that indicate which patient to offer a cholecystectomy or conservative treatment. Therefore, the indication to perform a cholecystectomy lies within the surgeons’ preference leading to variations in practice and consequently unnecessary cholecystectomies [8–11]. Annually, approximately 800,000 cholecystectomies are performed in the USA alone and the costs are estimated to be $6 billion [12]. Other developed countries show similar patterns of care. A systematic review reported in this journal demonstrated that up to 33 % of patients have persistent abdominal pain following cholecystectomy [13].

A strategy that is effective in selecting patients with abdominal pain and gallstones for surgery most likely to benefit from a cholecystectomy was developed. This strategy uses fixed selection criteria based on pain characteristics that are easy to use in clinical practice. Patients with abdominal pain and gallstones are selected for cholecystectomy if they fulfill one of the following two selection criteria [14, 15]: (1) episodic pain and (2) pain onset of 1 year or less before the outpatient clinic visit. Preoperative identification of patients with abdominal pain and gallstones who will benefit from a cholecystectomy from patients who will not will probably lead to more effective use of cholecystectomies, fewer surgery-related complications, and fewer unnecessary healthcare expenses. We performed a model-based economic evaluation to evaluate a strategy based on fixed criteria for selecting patients for a cholecystectomy against current practice.

## Materials and methods

**Fig. 1:**
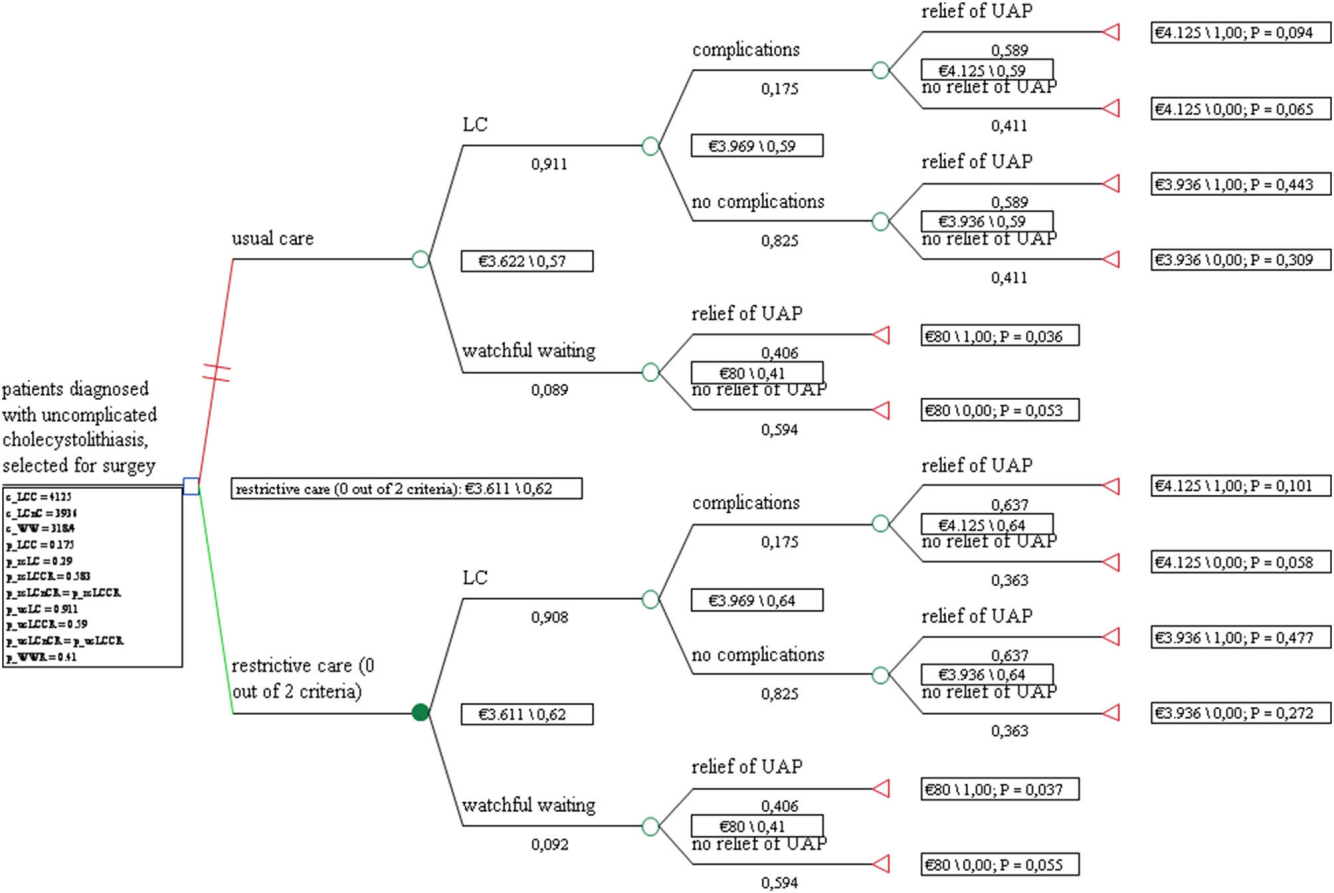
Decision model

The incremental cost-effectiveness of the new strategy, restrictive care, compared with the usual care strategy was analyzed, complying a healthcare perspective for a time horizon of one year. A decision analytic model was used with effectiveness expressed as absence of abdominal pain and costs in Euros (indexed to 2014). Models were built and analyzed using the decision analysis program TreeAge Software, Inc Williamstown, MA, USA, 2014 version (Fig. 1). The study was approved by the medical ethics committee, and informed consent for this modeling study was not needed.

### Cost-effectiveness model and model input

Cholecystectomy or watchful waiting was the treatment possibilities in both strategies. In the usual care strategy, the indication for cholecystectomy was left to the preference of the treating surgeon, thus without fixed selection criteria. In the restrictive care strategy, the indication for cholecystectomy was only made after meeting criteria as described above.

Table 1 shows the variables used as input and the specific data sources. A database of a prospective multicenter cohort study was used for the probability of getting a cholecystectomy [14]. In this database, all patients with cholelithiasis referred for cholecystectomy that visited the departments of surgery of one tertiary referral center and two non-academic teaching hospitals between June 2012 and June 2014 were recorded. The same prospective database was used for the probability of meeting the proposed criteria and the relief of upper abdominal pain afterward. The relief of pain after cholecystectomy in usual care was based on a systematic review [13]. Following a healthcare perspective, we only used direct medical costs for analysis. A previous study and existing guideline prices for the Netherlands were used to value an outpatient clinic visit, a cholecystectomy including an overnight stay, and surgical complications [16, 17]. Application of the criteria itself for cholecystectomy in the restrictive care strategy did not lead to additional costs in itself.

**Table 1:** Basic input variables and sources used in the decision model (shown in Fig. 1)

Input for the cost-effectiveness model	Data source
*Probability that a patient with abdominal symptoms and gallstones:* Satisfies one out of two criteria (thus receiving cholecystectomy) and satisfies two out of two criteria (thus receiving cholecystectomy)	Prospective multicenter cohort study [14] (306/337 = 0.908) (138/337 = 0.409)
Probability of receiving cholecystectomy in usual care strategy	Prospective database of a multicenter cohort study [14] (0.911)
Probability of having a complication of the surgery	Randomized controlled trial [17] (0.175)
Probability of having absence of pain after watchful waiting	Prospective study [26](0.41)
Probability of having absence of pain after cholecystectomy in usual care	Systematic review [13] (0.59)
*Probability that a patient has absence of pain after cholecystectomy in restrictive care:* Satisfies one out of two criteria and satisfies two out of two criteria	Prospective multicenter cohort study [14] (195/306 = 0.637) (100/138 = 0.725)
Costs of watchful waiting (= 1 extra outpatient clinic visit)	Cost-effectiveness guidelines [16] €314 ($354)
*Costs* Cholecystectomy including overnight stay, with or without complications, outpatient clinic visit	Randomized controlled trial and cost-effectiveness guidelines [16, 17] €4125 ($4645) with complications €3936 ($4432) without complications

For every modeling, study assumptions need to be made, which were the following in this study: Watchful waiting included an extra outpatient clinic visit made within 1 year in accordance with our clinical practice to reevaluate the patient’ symptoms. Additional diagnostic work-up is patient-dependent and only rarely applied and is therefore not included in this model [18].

### Analyses

The main outcome of both models was the incremental cost-effectiveness ratio expressed as the extra costs that need to be invested in order to get one more patient with relief of abdominal pain. Two analyses were performed. The first analysis focused on the incremental cost-effectiveness of the new strategy in gallstone patients having a cholecystectomy if one of two criteria would be fulfilled compared with usual care. The second analysis focused on the incremental cost-effectiveness of the new strategy in gallstone patients having a cholecystectomy if two out of two criteria would be fulfilled. Models were analyzed using a probabilistic sensitivity analysis. With this analysis, the model runs a 1000 times, each time picking another value from the distribution underlying the input parameters. Beta distributions for the probabilities of getting a cholecystectomy and relief of abdominal pain were used. For the cost parameters, however, no data were available to construct a distribution. Results from the 1000 runs are graphically presented as scatter plots on cost-effectiveness planes and as means with 95 % percentiles.

## Results

**Fig. 2:**
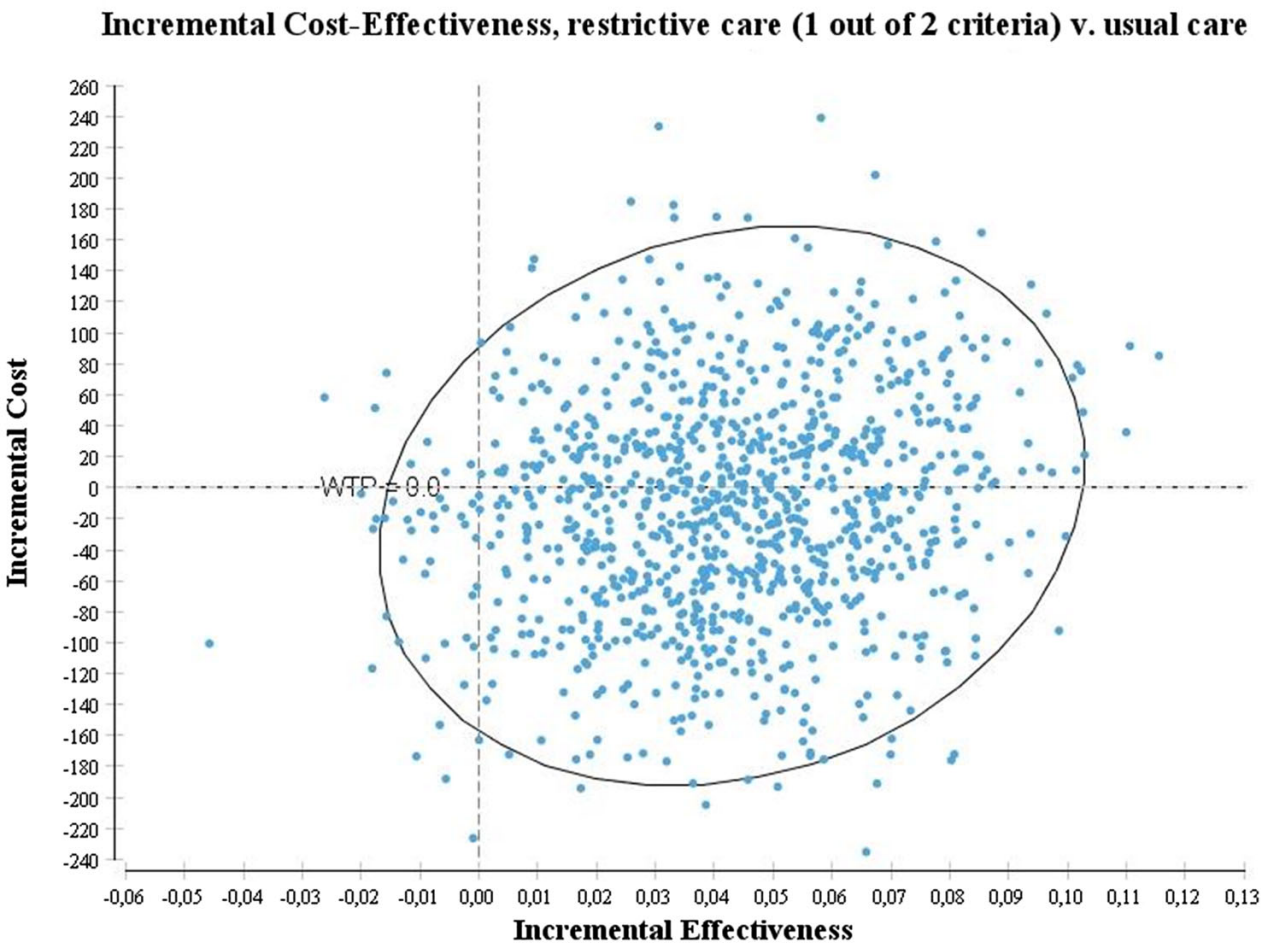
Probabilistic sensitivity analysis of the model with the new strategy of gallstone patients having cholecystectomy if one out of two criteria have been satisfied

The results of the probabilistic sensitivity analysis of the first decision model with the new strategy of gallstone patients having a cholecystectomy if one of two criteria has been satisfied are shown in Fig. 2. The new strategy was more effective compared with the usual care strategy and also less expensive. The mean percentage of patients with absence of pain in the new strategy was 62 % (95 % percentile 0.57–0.66), whereas with the usual care strategy this was 57 % (95 % percentile 0.55–0.60). The costs of the new strategy were €3610 (95 % percentile 3487–3722) ($4065; 95 % percentile 3927–4191), whereas the costs of the usual care strategy was €3622 (95 % percentile 3536–3706) ($4078; 95 % percentile 3982–4173). The mean cost difference was −€12 (95 % percentile −134–105) (−$14; 95 % percentile −151–118) with a mean effectiveness difference of 4.0 (95 % percentile 0.2–8.0) for the new strategy compared with the usual care strategy. Fifty-three percent of the simulations are located in the dominant quadrant, meaning a higher percentage of patients with relief of upper abdominal pain against lower costs. The 95 % upper limit of the incremental cost-effectiveness ratio (ICER) is €4114 ($4633) per extra patient with relief of upper abdominal pain. This implies that €4114 ($4633) needs to be paid to relieve one extra patient from his abdominal pain in this model.

**Fig. 3:**
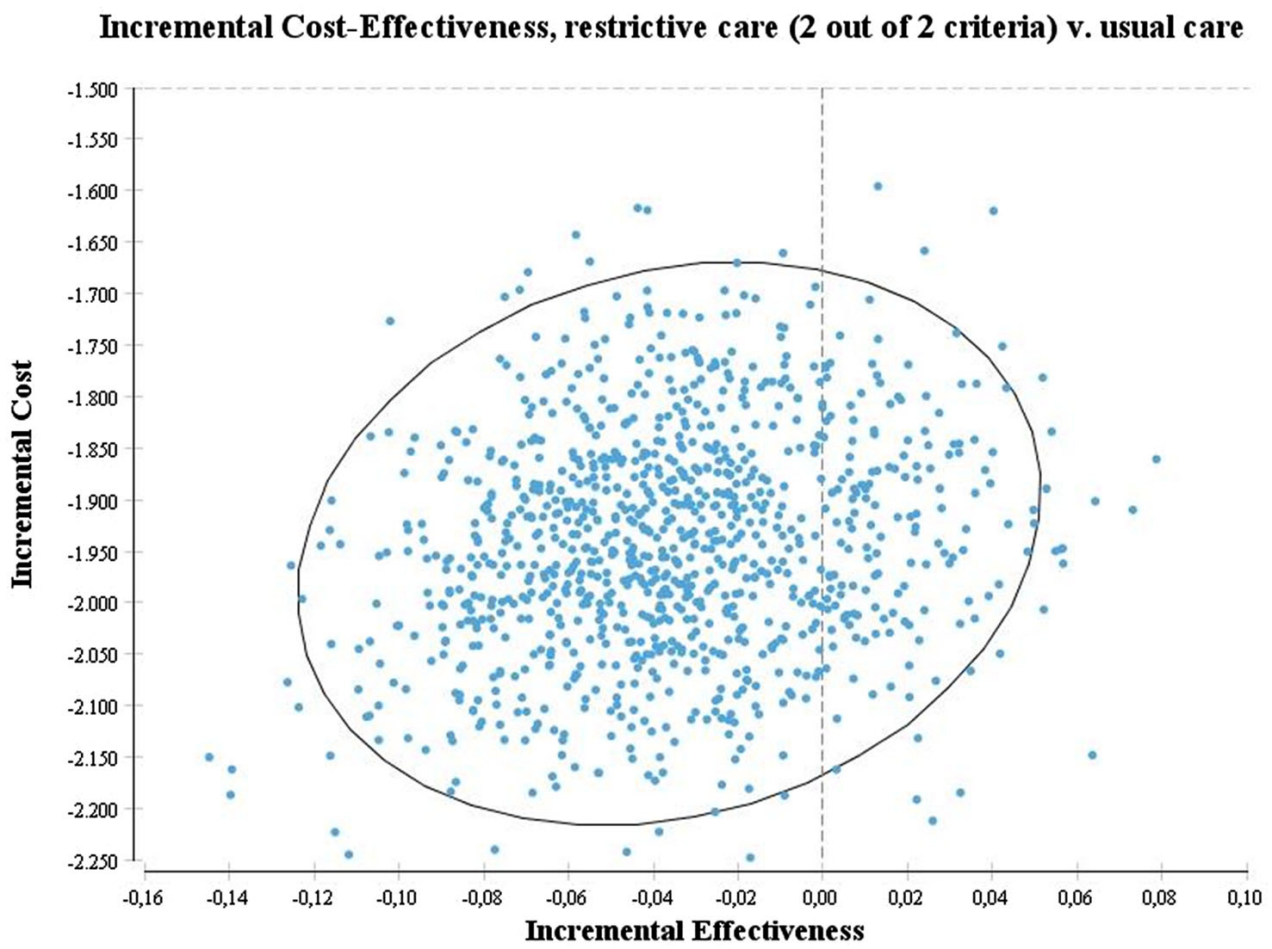
Probabilistic sensitivity analysis of the model with the new strategy of gallstone patients having cholecystectomy if two out of two criteria have been satisfied

The results of the probabilistic sensitivity analysis of the second model with the new strategy of gallstone patients having a cholecystectomy if both criteria have been satisfied are shown in Fig. 3. The mean percentage of patients with absence of pain in the new strategy was 54 % (95 % percentile 0.46–0.61), whereas with the usual care strategy this was 57 % (95 % percentile 0.55–0.60) and therefore more effective. The cost of the new strategy was €1675 (95 % percentile 1471–1886) ($1886; 95 % percentile 1656–2124), whereas the cost of the usual care strategy was €3618 (95 % percentile 3527–3700) ($4074; 95 % percentile 3972–4167). While in all simulations the new strategy resulted in lower costs, only 16 % of the simulations resulted in a higher percentage of patients with relief of upper abdominal pain.

## Discussion

This study showed that the strategy of using strict selection criteria may be a cost-effective method to reduce the proportion of patients with pain after cholecystectomy. The strategy of applying one out of two criteria resulted in a 4 % higher mean proportion of patients with absence of pain compared to current practice with similar costs. The majority (54 %) of the simulations resulted in the restricted care being dominant, meaning less expensive and more effective. In those simulations, the restrictive care strategy was more effective but also more expensive, and there was a fair maximum to be paid per extra patient with relief of abdominal pain. Application of the more stringent two out of two criteria resulted in a 3 % lower mean proportion of patients with absence of pain, but against lower costs.

In a previous study, we showed that duration of pain of 1 year or less has a slightly higher odds ratio for absence of pain after cholecystectomy as compared to episodic abdominal pain (2.22 vs. 2.13), although this difference is not significant [14]. Altogether we would recommend to implement the strategy of applying one out of two criteria. Application of these criteria would offer surgeons less room for personal preferences which patient to offer a cholecystectomy and which patient to treat conservatively. This strategy would therefore provide a tool for better patient selection for each treatment arm. A recent cost-effectiveness study reported in this journal comparing cholecystectomy with observation for uncomplicated symptomatic cholecystolithiasis or acute cholecystitis reported that cholecystectomy is the preferred treatment for symptomatic gallstones. On average, surgery costs £1236 (€1448; $1631) more per patient than conservative management, but was more effective. However, the study also reported that 55 % of the patients randomized to the observation group did not require surgery indicating that it may be a valid alternative to surgery [19]. Application of fixed criteria for cholecystectomy may increase the cost-effectiveness of cholecystectomy and conservative treatment as shown in this study.

Effectiveness of an intervention is often reported in cost-effectiveness studies as quality-adjusted life years (QALY) [19]. However, abdominal pain is the most characteristic feature of uncomplicated symptomatic cholecystolithiasis and therefore the main aim of cholecystectomy in this patient group [3–6]. In addition, absence of pain after cholecystectomy is the main predictor of a patient-reported successful outcome of the operation [20]. The Gastrointestinal Quality of Life Index, a patient-reported outcome measure computing quality of life, may not be sufficiently disease-specific [21, 22]. Abdominal pain, for example, is equally scored as flatulence in this questionnaire. Other patient-reported outcome measures computing quality of life are not different in weighing persistent abdominal pain [23]. Furthermore, a quality of life score is less applicable in surgical practice compared to presence or absence of abdominal pain. We therefore selected absence of postoperative abdominal pain as effectiveness outcome.

This study must be considered within the context of some limitations. First, the criteria for selection for cholecystectomy remain non-specific, although they are more specific than current practice entirely based on the surgeons’ preference. Second, the criteria of the new strategy were not externally validated, although this may have been challenging to perform due to strong treatment preferences of patients and surgeons [24–26]. Third, we focused on uncomplicated symptomatic cholelithiasis patients. Patients with complicated symptomatic cholelithiasis were not considered. Exclusion of complicated symptomatic cholelithiasis may not have had a large impact as the patient group with uncomplicated symptomatic cholelithiasis only have an annual 1–3 % risk on complications because of the stones [27]. Furthermore, the observation group of a randomized controlled trial of patients with uncomplicated symptomatic cholecystolithiasis did not suffer complications during 14 years of follow-up [28]. Fourth, we excluded patients having a bile duct injury as this specific complication of cholecystectomy has a low incidence of 0.04–1.5 % [29, 30]. Fifth, we did not consider additional diagnostic work-up because of lack of data, variability, and patient-dependency [18]. Finally, the new strategy was evaluated from a healthcare perspective for a time horizon of 1 year. If a societal perspective would be taken into account, the restrictive care strategy of having one out of two criteria satisfied would probably be even more cost-effective, because this strategy was more effective in terms of relief of abdominal pain and it prevented cholecystectomies, probably preventing sick leave. Especially, patients with ongoing abdominal pain after cholecystectomy would continue to seek medical help with additional diagnostic interventions.

This study should be considered a pilot study before assessing the cost-effectiveness of the application of these criteria in an actual trial. Apart from confirming these results in a prospective randomized multicenter study, future research should focus on further maximizing the cost-effectiveness of cholecystectomy. Determination of patients with cholelithiasis at risk for complications due to the gallstones may benefit from earlier cholecystectomy. Selection for earlier surgery of those patients who are most likely to benefit will further increase the cost-effectiveness of this common surgical procedure. In addition, not only should be assessed which patient will benefit from cholecystectomy, but also which patient will benefit most. Episodic abdominal pain due to gallstones may not significantly affect the health status of all patients to that extent that a cholecystectomy is required. The necessity may depend on frequency, duration, and intensity of the abdominal pain episodes [31]. Reliable prediction models combining clinical parameters with patient-reported outcome measures may facilitate efficient use of scarce healthcare resources [32].

In conclusion, the new strategy was more effective, against similar costs, than current practice if one out of two criteria were applied. More stringent application of criteria resulted in loss of effectiveness. The new strategy of using strict selection criteria may be a cost-effective method to reduce proportion of patients with pain after cholecystectomy.
